# Evaluating the changes in household purchases of foods and drinks high in fat, salt and sugar following Bristol’s outdoor advertisement restrictions policy: a quasi-experimental study

**DOI:** 10.1136/bmjph-2025-004137

**Published:** 2026-04-30

**Authors:** Genevieve Buckland, Carlos Sillero-Rejon, Zoi Toumpakari, Russ Jago, Amy Yau, Steven Cummins, James Nobles, Sarah Kate Harding, Jeremy Horwood, Agnes Nairn, William Hollingworth, Sarah Blake, Frank de Vocht

**Affiliations:** 1 Centre for Public Health, University of Bristol, Bristol, UK; 2 NIHR Applied Research Collaboration West, Bristol, UK; 3 Population Health Sciences, University of Bristol, Bristol, UK; 4 Centre for Exercise, Nutrition and Health Sciences, University of Bristol, Bristol, UK; 5 NIHR ARC West, Bristol, UK; 6 Population Health Innovation Lab, London School of Hygiene & Tropical Medicine, London, UK; 7 Obesity Institute, School of Health, Leeds Beckett University, Leeds, UK; 8 Bristol Hub for Gambling Harms Research, University of Bristol, Bristol, UK; 9 Centre for Research in Health and Social Care, University of Bristol, Bristol, UK

**Keywords:** Public Health, Obesity, Food Quality

## Abstract

**Introduction:**

In 2021, Bristol (UK) became the first city outside London to introduce a policy restricting advertisements of high fat, salt and sugar (HFSS) products and other unhealthy commodities (alcohol, gambling and payday loans) on council-owned advertisement sites, predominantly bus stops. This research evaluates the impact of this policy on household purchasing of HFSS products specifically.

**Methods:**

Take-home purchasing data for food and drink items recorded from October 2020 to May 2023 (74 weeks before and 59 weeks after policy implementation) by 1012 households from Kantar’s Worldpanel division were included in the analysis: 217 households from Bristol (intervention) and 795 from Cardiff, Sheffield, Gloucestershire and South Gloucestershire (controls). Controlled interrupted time-series analyses adjusted for confounders were used to estimate the change in average weekly household purchases of energy and nutrients from HFSS products in the post-intervention period (from 1 April 2022) compared with the counterfactual (data from controls and pre-intervention in Bristol).

**Results:**

There was no indication of changes in purchasing of HFSS products in Bristol following the policy; mean change in weekly household purchases of energy and nutrients from take-home HFSS products was 897.6 kcal (95% CI −57.7 to 1.853) or 6.1% (95% CI −0.7 to 12.9) for energy, 4.6% (95% CI −2.6 to 11.9) for fat, 6.1% (95% CI −2.9 to 15.0) for sugar and −1.9% (95% CI −17.4 to 13.6) for salt, respectively, compared with the counterfactual. Stratified analyses by household socio-demographics also showed no evidence of changes in energy purchased from HFSS products in Bristol.

**Conclusions:**

This study did not observe evidence of changes in purchasing of HFSS products following Bristol’s advertising restriction policy. Bristol City Council owns a relatively small part (~30%) of the city’s outdoor advertisement estate, so changes in exposure may have been too small to lead to measurable effects. Extending similar policies to cover more outdoor spaces and advertising platforms is probably required to impact purchasing behaviour.

**WHAT IS ALREADY KNOWN ON THIS TOPIC**

- Few studies have evaluated the purchase-related impact of policies restricting outdoor advertising of unhealthy foods and drinks.
- The main evidence to date comes from an evaluation of the Transport for London advertising restriction policy, which showed the policy was associated with reductions in household purchases of energy, sugar and fat from high fat, salt and sugar (HFSS) products.

**WHAT THIS STUDY ADDS**

- Our study helps understand the effect on purchasing of HFSS products following the implementation of an outdoor advertising restrictions policy in Bristol, a UK city where a lower proportion of outdoor advertising space was affected compared with London.
- The reduction in HFSS food advertising resulting from Bristol’s advertising restrictions policy may have been too small to result in a measurable impact on HFSS purchasing behaviour.

**HOW THIS STUDY MIGHT AFFECT RESEARCH, PRACTICE OR POLICY**

- This study adds to the evidence which can be used by governments and local authorities when designing similar policies to improve public health.
- Future policies implemented in similar settings may benefit from additional complementary advertising restriction agreements or legislations covering more outdoor spaces in order to significantly impact purchasing behaviour.

## Introduction

 The global increase in consumption of unhealthy processed foods, generally high in fat, salt and sugar (HFSS), is considered an important factor contributing to the rising trend in non-communicable diseases (NCD) such as obesity, type II diabetes and cardiovascular disease.^1^ In the UK in 2025, obesity and excess weight were estimated to cost the economy and wider society £126 billion, with £12.6 billion due to the National Health Service (NHS) costs.^2^ The commercial determinants of health include the strategies and approaches used by the private sector to promote products which negatively affect people’s health.^3^ Such strategies involve marketing of HFSS products, which can influence attitudes, preferences, purchasing and consumption of these foods and drinks,^4 5^ thereby contributing to obesity and diet-related diseases.^5 6^

International health agencies and public health advocacy organisations are increasingly calling on governments to restrict unhealthy food advertising due to their negative impact on eating behaviours.^3 7^ Accordingly, many governments now have mandatory regulations in place to restrict the marketing of HFSS products across a variety of media, particularly traditional broadcast media advertising targeted at children.^8 9^ Nevertheless, people continue to be exposed to marketing of unhealthy foods and drinks in other settings, including outdoor public spaces, such as advertising on digital or poster boards, billboards and hoardings, where HFSS advertising is abundant and can impact food purchasing behaviours.^10 11^ However, new policies are increasingly being implemented to restrict advertising of unhealthy foods/drinks, and often other unhealthy commodities, specifically in outdoor publicly owned sites.^10 12^

In the UK, no national regulations exist restricting marketing of HFSS products in outdoor spaces. These advertising sites can have considerable impact on total exposure to HFSS products, since outdoor advertising is estimated to reach 98% of the UK population,^13^ and a 2021 study in Scotland reported that approximately 33% of bus stop advertisements were for unhealthy foods and drinks.^14^ A sizeable proportion of outdoor advertising space in the UK is owned by government local authorities (LAs), which may be used to advertise HFSS products.^15^ However, such advertising content goes against public health initiatives by the same LAs aimed at improving population health and reducing social gradients in health.

UK LAs have substantial legislative powers to restrict commercial advertising and sponsorship of unhealthy commodities such as less healthy foods/drinks, but also tobacco, alcohol and gambling, on their council-owned advertising spaces. A review of advertising restriction policies across English LAs in 2023 found that approximately a third (n=106) had such a policy (which generally covered all types of outdoor advertising), and 24% of these (n=25) included an explicate reference to less healthy foods.^16^ Despite growing momentum for introducing local policies restricting advertising of HFSS products on council-owned outdoor spaces in England,^15^ few studies (nationally or globally) have evaluated the effectiveness of these policies.^12 15^

The main evidence in the UK comes from an evaluation of the pioneering 2019 Transport for London (TfL) policy, which restricted advertisements of HFSS products across the entire TfL network. This public health initiative was associated with a relative reduction in average weekly household purchases of energy from HFSS products of 6.7% (−1001kcal; 95% CI −1546 to −456) with the largest reductions from chocolate and confectionery.^17^ Additional benefits from modelling calculations estimated that after 3 years the TfL policy would result in 16 394 additional quality-adjusted life years and save £218 million in NHS and social care costs.^18^

In 2021, Bristol became the first city outside of London to introduce a policy to restrict advertisements of HFSS products, along with other unhealthy commodities including alcoholic drinks, gambling and payday loans, on their council-owned outdoor spaces, as detailed in their Advertising and Sponsorship Policy.^19^ The Bristol Evaluation of Advertising Restrictions (BEAR) study uses a mixed-method design to evaluate the implementation, effectiveness and impact of the policy.^20^,^22^ This arm of the evaluation study aims to assess whether the implementation of Bristol’s advertising restrictions policy is associated with changes in household HFSS food and drink purchases.

## Material and methods

The study protocol was published prior to the start of the analyses and is available from Open Science Framework (https://osf.io/s6p57). The study received ethical approval by the University of Bristol Faculty of Health Sciences Research Ethics Committee (FREC), reference 17 695. All study panel participants have given consent for their data to be used. Results are reported in accordance with STROBE guidelines (online supplemental material methods (OSM M1)).

### Study design

In November 2021, Bristol City Council introduced an Advertising and Sponsorship policy which restricted the advertisements of HFSS products, alcoholic drinks, gambling and payday loans on council-owned bus stops and other advertising spaces.^19^ A controlled interrupted time series (CITS) evaluation design was used for this natural experiment to estimate if any changes following the policy in household purchases of HFSS products in Bristol were associated with the policy. Weekly household purchases were obtained for households in Bristol (intervention area) and three control areas (Cardiff, Sheffield and South Gloucestershire and Gloucestershire), selected for specific geographical and sociodemographic reasons (OSM M2) and which had no similar restrictions on outdoor advertising throughout the study period. The study power was calculated using data from the TfL evaluation and was powered to detect a minimal effect change of 8.7% in weekly household mean energy purchased from HFSS products.

### Outcome data

The study uses take-home (TH) and out-of-home (OOH) data on food and drink purchases from 1101 households from Kantar Worldpanel’s GB shopper panels (OSM M2). Kantar Worldpanel maintains a nationally representative live panel of households who provide item-level data on their day-to-day food and beverage purchases, collected using handheld barcode scanners, while non-barcoded products, such as loose fruits and vegetables, are recorded using bespoke barcodes. Food and drink data were collected over the period of 74 weeks before to 59 weeks after the policy was implemented (from 2020 to 2023, covering 133 weeks).

Kantar provides information on the total weight and energy (KJ), sugar (g), sodium (g), saturated fat (g), protein (g) and fibre (g) content of each household purchase, along with the product’s Nutrient Profiling Model score^23^ (OSM M2) and HFSS classification based on the Food Standards Agency methodology,^23^ the same methods used to determine if the food and drink advertisements are subject to Bristol advertisement restriction policy. For OOH purchases, nutritional information was not available for 84% of items, so their HFSS status was estimated based on market sector category and product name (ie, if it fell within one of the key HFSS categories), and they were analysed as packs. A pack was an individual item scanned and could be a single serving or a multipack and so did not reflect the volume purchased. TH and OOH purchase data were separately aggregated into total weekly household purchases of HFSS products (overall and by nutrient and food group) and non-HFSS products.

### Outcomes

The primary outcome was the relative change in weekly household TH purchases of energy, fat, saturated fat, sugar and salt from HFSS products in the intervention group compared with the counterfactual. Additional subgroup analyses assessed if any changes in these HFSS purchases were moderated by household sociodemographic characteristics: body mass index (BMI), age of main shopper and children in the household. Secondary outcomes included changes in TH energy and nutrients from five main HFSS categories: chocolate and confectionery, puddings and biscuits, sugary drinks, sugary cereals and savoury snacks. Relative changes in energy and nutrients from OOH purchase of HFSS products were also assessed.

### Statistical analysis

All data were analysed using STATA (version 18). Household characteristics of the intervention and control groups were compared to assess potential differences. CITS were used to estimate the relative mean difference in energy and nutrients from HFSS products in the intervention group (Bristol post-policy period) compared with the counterfactual scenario—the theoretical trend in household purchasing in Bristol if the intervention had not been implemented. The counterfactual was created by extrapolating the pre-intervention trend in the intervention group combined with the post-intervention changes in the control group. The CITS model included an indicator variable for intervention versus control areas, weeks elapsed since study start, an indicator signifying pre/post-intervention periods and their interactions. Separate CITS models were used for each analysis of primary and secondary outcomes. Although the policy was introduced in November 2021, the intervention date was defined as the first week of April 2022 (week 75), which was when the policy came into effect because existing advertising contracts had all ended. All CITS models were adjusted for age of main food shopper, number of adults and children in the household, main festivals, seasons, socioeconomic position, BMI and ethnicity of main shopper (OSM M3). Socioeconomic position was categorised according to the National Readership Survey (NRS) occupational social grade classes and grouped into low (D, E), middle (C1, C2) and high (A, B).^24^

The dataset contained zero values because not all households purchased HFSS products every week (ranging from 35% for total HFSS products to 68.1% for sugary cereals). Therefore, a two-part CITS model for mixed discrete and continuous outcomes was used to account for zero-inflation; part 1 (logit) to estimate the probability of purchasing a product and part 2 (generalised linear model) to estimate how much was purchased, if a product was purchased.

### Sensitivity analyses

In sensitivity analyses, all models were repeated with each control area separately, as well as without control areas. A further sensitivity analysis by reporter status (regular vs irregular) explored the influence on the results of potential incomplete reporting. Regular reporters were defined as households that reported any food or drink in at least 90% of the study weeks. An additional analysis included further adjustment for COVID-19 restrictions, defined as an indicator variable for all weeks until the staged exit from the last COVID lockdown (end of March 2021). Finally, we assessed changes in mean weekly household purchases of non-HFSS products to evaluate if the Bristol advertising policy had any potential unplanned influence on other products.

## Results

**Figure 1:**
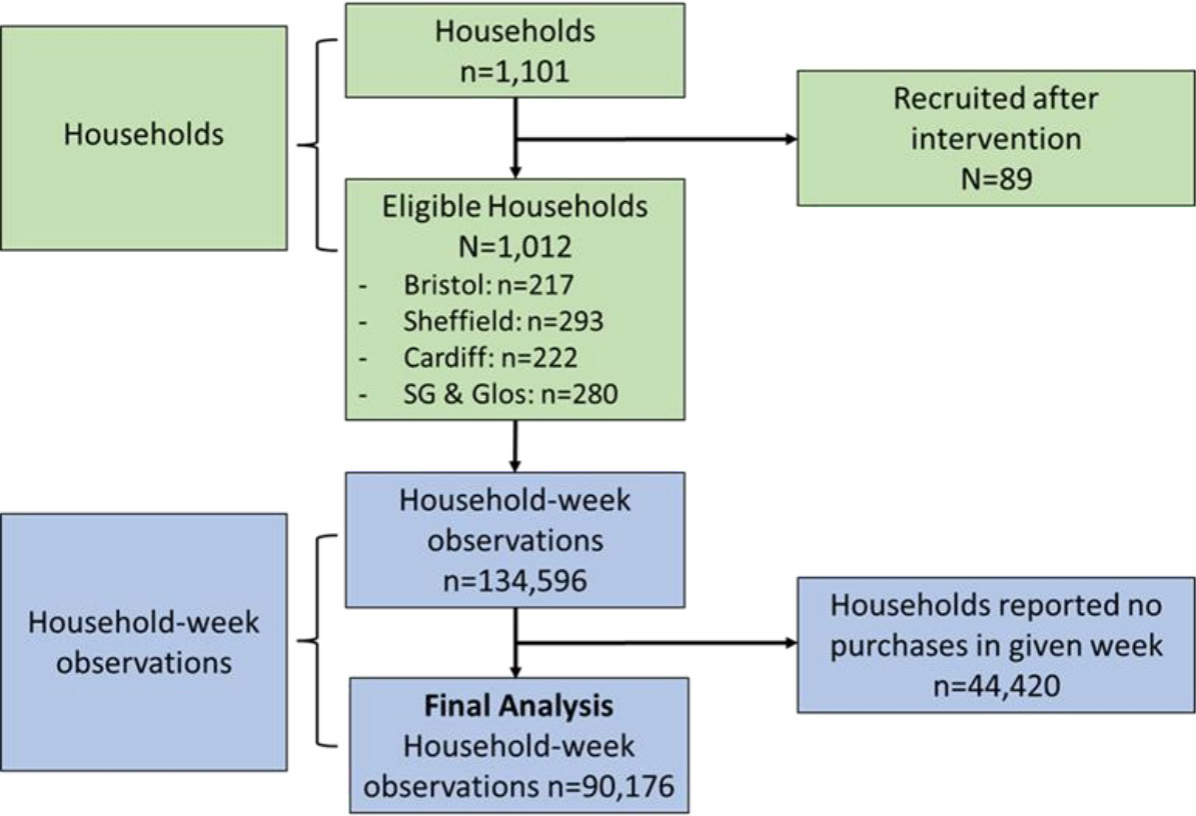
Study flow diagram detailing eligibility and inclusion of households and household weekly take-home purchasing data from Kantar’s Worldpanel division.

Of the initial 1101 households providing TH purchase data, 89 were excluded because their purchasing records began after the intervention. The majority of households did not report in all 133 study weeks; n=44 420 (33%) of household week observations were empty; and mean reporting was 89.1 weeks (SD 42.0 (median 105 weeks (IQR 59–125))). 1012 households were included in the final TH analysis (figure 1), with a total of 90 176 weeks of household purchasing data spanning from October 2020 to May 2023. Similar summary statistics are provided for OOH purchasing (163 households) in OSM Figure(F)1.

Sociodemographic characteristics of the households in the intervention (n=217) and control (n=795) areas were generally comparable, although there were more high socio-economic status (SES) households in the control areas (table 1 and OSM T1). Household characteristics of each of the control areas providing TH data and of the subgroup of households providing OOH data are detailed in OSM T1 and OSM T2, respectively.

**Table 1:** Descriptive characteristics of the intervention households (from Bristol) and control households, using Kantar’s Worldpanel division take home purchasing data

Characteristics	Subcategory	Total (n=1012)	Intervention (n=217)	Control (n=795)	P value*
Household characteristic					
No. of adults in the household, mean (SD)		2.1 (0.9)	2.0 (0.8)	2.2 (0.9)	0.02
No. of children in the household, mean (SD)		0.6 (1.0)	0.7 (1.0)	0.6 (1.0)	0.30
Children in the household, n (%)	Yes	371 (36.7)	88 (40.6)	283 (35.6)	
	No	641 (63.3)	129 (59.4)	512 (64.4)	0.18
Main food shopper characteristics					
Age (years), mean (SD)		51.5 (15.1)	50.2 (14.9)	51.8 (15.1)	0.17
Socioeconomic position, n (%)	High	204 (20.2)	35 (16.1)	169 (21.3)	
	Middle	603 (59.6)	134 (61.8)	469 (59.0)	0.23
	Low	205 (20.3)	48 (22.1)	157 (19.7)	
Body mass index, n (%)	Not overweight	313 (30.9)	68 (31.3)	245 (30.8)	
	Overweight/obese	514 (50.8)	106 (48.9)	408 (51.3)	
	Unknown	185 (18.3)	43 (19.8)	142 (17.9)	0.75
Ethnicity, n (%)	White British	837 (82.7)	177 (81.6)	660 (83.0)	
	White Other	61 (6.0)	14 (6.5)	47 (5.9)	
	Non-White	61 (6.0)	11 (5.1)	50 (6.3)	0.57
	Missing/Unknown	53 (5.2)	15 (6.9)	38 (4.8)	

* Differences tested using chi-squared for categorical variables and t-test for continuous variables.

In total, 3 629 732 TH food and drink items were purchased over the study period, of which 1 405 353 (39%) were classified as HFSS (data not tabulated). Mean weekly household energy (kcal) and nutrients (g) purchased from HFSS products were on average higher in the control areas compared with the intervention area, with the exception of sugar, and were also slightly higher in the pre-policy compared with the post-implementation period (table 2). A similar pattern was observed for energy purchased from HFSS food groups (OSM T3).

**Table 2:** Unadjusted weekly household mean energy purchased from high fat, salt and sugar (HFSS) products and non-HFSS products and mean grams of fat, saturated fat, sugar and sodium from HFSS products purchased in pre- and post-intervention periods in the intervention and control group, using Kantar’s Worldpanel division take-home purchasing data (October 2020 to May 2023)

Study time period and food/drink category	Weekly household mean purchases, mean (SD)		
	Total (n=1012)	Intervention (n=217)	Control (n=795)*
Pre-intervention period			
All food/drink (kcal)	32 562.4 (25 765.8)	30 982.1 (22 954.5)	32 996.3 (26 469.4)
HFSS products (kcal)	16 868.7 (14 452.8)	16 757.0 (15 083.2)	16 899.4 (14 274.8)
Non-HFSS products (kcal)	15 692.2 (16 744.7)	14 224.9 (10 871.2)	16 095.1 (18 004.9)
Fat from HFSS products (grams)	960.9 (939.1)	933.1 (912.0)	968.5 (946.2)
Saturated fat from HFSS products (grams)	401.4 (369.2)	387.1 (374.7)	405.3 (367.5)
Sugar from HFSS products (grams)	905.4 (1058.4)	913.9 (1129.8)	903.1 (1037.9)
Salt from HFSS products (grams)	60.5 (137.0)	58.3 (127.5)	61.1 (139.4)
Post-intervention period			
All food/drink (kcal)	30 082.4 (27 474.2)	28 437.6 (21 305.4)	30 539.9 (28 941.3)
HFSS products (kcal)	15 190.1 (13 360.7)	15 147.6 (14 005.3)	15 201.9 (13 176.0)
Non-HFSS products (kcal)	14 891.1 (20 747.8)	13 290.0 (10 049.4)	15 336.4 (22 830.3)
Fat from HFSS products (grams)	872.5 (873.4)	852.9 (855.9)	877.9 (878.2)
Saturated fat from HFSS products (grams)	362.0 (338.9)	349.9 (343.0)	365.3 (33.7)
Sugar from HFSS products (grams)	809.7 (967.3)	822.5 (1073.6)	806.1 (935.5)
Salt from HFSS products (grams)	54.0 (124.3)	53.7 (129.0)	54.1 (122.9)

* Difference in mean of purchased nutrients between intervention and control groups tested using Kruskal-Wallis H test (due to non-normal distribution of data). All p values <0.01.

**Figure 2:**
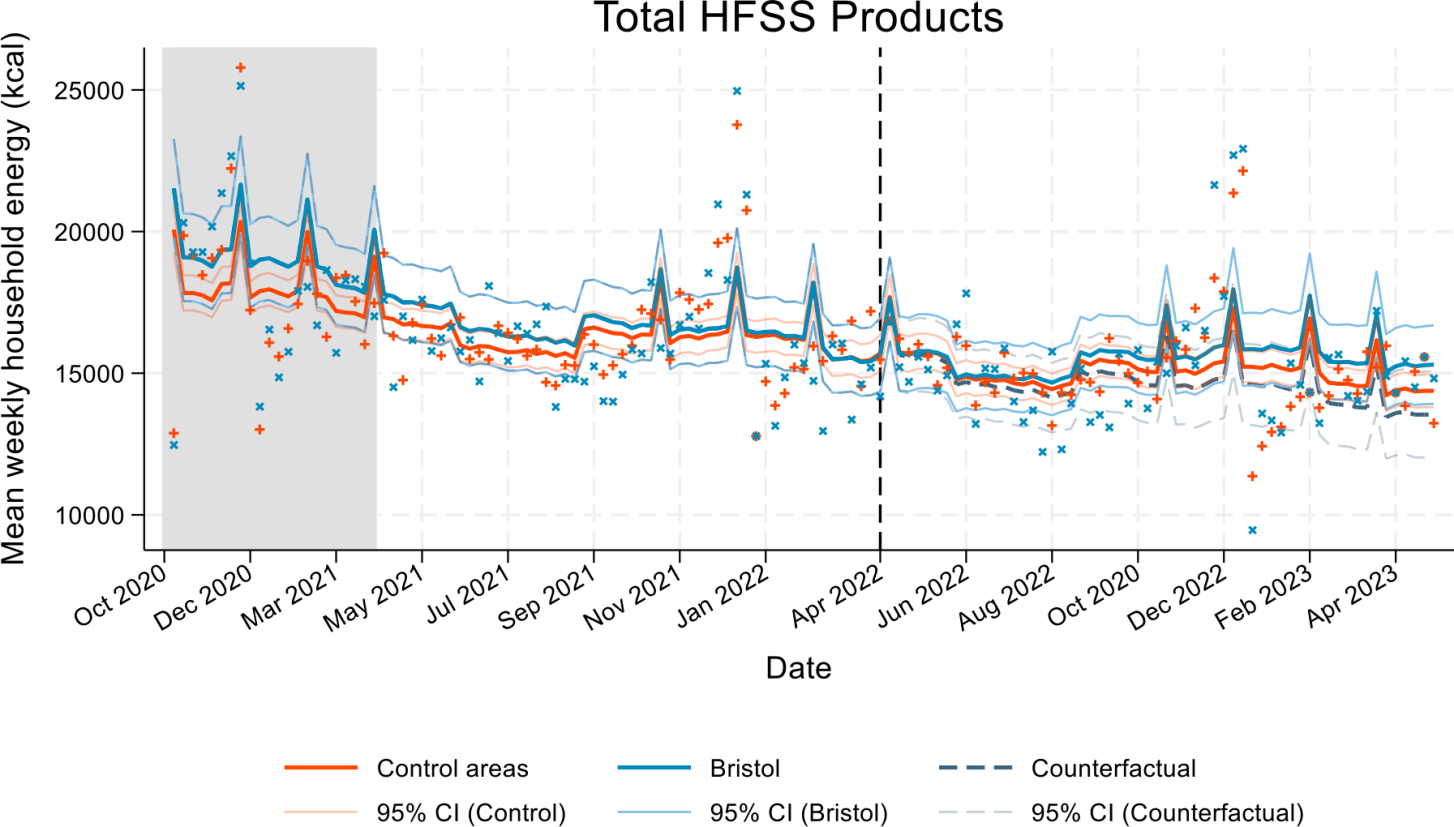
Adjusted weekly household mean energy purchased from all HFSS products in Bristol (intervention), Cardiff, Sheffield, Gloucestershire and South Gloucestershire (control), and the counterfactual. Vertical line=date of intervention implementation. Grey shaded area=period up until end of last COVID lockdown staged exit (end of March 2021). Blue x and red + indicate data points for Bristol and control areas, respectively. Data period = October 2020 to May 2023. Spikes represent festival weeks included in the adjusted models. Numerical values linked to this graph are detailed in table 3. HFSS, high fat, salt and sugar.

Our results showed no evidence of a change in weekly household purchases of energy from TH HFSS products following the implementation of the policy; mean kcal change and percentage change was 897.6 kcal (95% CI −57.7 to 1853.0) and 6.1% (95% CI −0.7 to 12.9) respectively, in Bristol compared with the counterfactual (table 3; figure 2). Similarly, there was no evidence of changes in nutrients from HFSS products; the mean percentage change in weekly household purchases of nutrients from HFSS products in Bristol compared with the counterfactual was 4.6% (95% CI −2.6 to 11.9) for fat, 7.1% (95% CI −1.1 to 15.3) for saturated fat, 6.1% (95% CI −2.9 to 15.0) for sugar and −1.9% (95% CI −17.4 to 13.6) for salt (table 3 and OSM F2). We also did not observe changes in energy or nutrients from key HFSS food groups (table 3 and OSM F3). Although there was an increase in weekly household purchases of energy from sugary drinks in the intervention compared with the counterfactual (61.4 kcal; 95% CI 4.8 to 118.0), this was not evident in the percentage change (34.9%; 95% CI −8.0 to 77.8) (table 3).

**Table 3:** Adjusted changes and percentage changes in weekly household mean (95% CI) energy and nutrients purchased from high fat, salt and sugar (HFSS) products, overall and by HFSS food categories, in Bristol (intervention group) compared with the counterfactual*, October 2020 to May 2023 (n=1012), based on Kantar’s Worldpanel division take home purchase data

Nutrient	Total HFSS products	Chocolate and confectionery	Puddings and biscuits	Sugary drinks	Sugary cereals	Savoury snacks
Energy						
Kilocalories	897.6 (-57.7, 1853.0)	192.6 (-31.2, 416.5)	303.2 (-37.6, 644.0)	61.4 (4.8, 118.0)	80.2 (-31.9, 192.4)	118.5 (-38.4, 275.3)
Percent	6.1 (-0.7, 12.9)	13.7 (-3.9, 31.3)	9.9 (-2.2, 22.0)	34.9 (-8.0, 77.8)	31.2 (-25.8, 88.1)	9.8 (-4.4, 23.9)
Fat						
Grams	39.0 (-20.1, 98.1)	8.3 (-1.9, 18.5)	11.0 (-4.6, 26.6)	1.4 (0.2, 2.6)	1.2 (-1.9, 4.3)	6.4 (-2.3, 15.1)
Percent	4.6 (-2.6, 11.9)	14.0 (-5.2, 33.1)	8.1 (-4.2, 20.5)	65.9 (-26.5, 158.4)	20.7 (-43.1, 84.4)	9.6 (-4.7, 23.8)
Saturated fat						
Grams	24.0 (-2.2, 50.3)	5.2 (-0.3, 10.7)	9.2 (1.3, 17.1)	1.2 (0.2, 2.1)	0.6 (-0.4, 1.4)	1.0 (-0.8, 2.9)
Percent	7.1 (-1.1, 15.3)	16.1 (-3.1, 35.2)	14.1 (0.6, 27.6)	73.1 (-30.5, 176.7)	30.3 (-38.4, 99.0)	9.5 (-8.8, 27.8)
Sugar						
Grams	48.0 (-19.8, 115.7)	22.1 (-5.6, 49.7)	17.6 (-11.4, 46.5)	8.6 (-1.4, 18.6)	4.1 (-2.7, 10.9)	0.1 (-2.4, 2.6)
Percent	6.1 (-2.9, 15.0)	13.1 (-5.0, 31.2)	7.1 (-5.4, 19.5)	27.5 (-13.4, 68.4)	24.7 (-25.3, 74.7)	1.1 (-22.9, 25.1)
Salt						
Grams	−1.1 (-10.1, 7.9)	−0.01 (-0.2, 0.2)	0.3 (-0.1, 0.8)	0.1 (-0.1, 0.3)	0.1 (-0.1, 0.3)	0.5 (0.1, 1.0)
Percent	−1.9 (-17.4, 13.6)	−1.4 (-23.4, 20.7)	10.5 (-5.4, 26.5)	25.1 (-40.2, 90.3)	25.8 (-34.8, 86.3)	15.6 (0.4, 30.8)

Weekly household mean purchases were estimated from a controlled interrupted time series two-part model: part 1 (logit) and part 2 (generalised linear model), with gamma distribution for energy and nutrients and negative binomial distribution for packs. Models were adjusted for festivals, season, number of adults in household, number of children in household, age, and socioeconomic position of main food shopper, BMI and ethnic group. Cluster-robust standard errors were used. Household-week observations where households did not report any food and drink purchases that week were dropped. Data period = October 2020 to May 2023.

* Counterfactual: the theoretical trend in household purchasing in Bristol if the intervention had not been implemented, calculated by extrapolating the pre-intervention trend in the intervention group combined with the post-intervention changes in the control group.

BMI, body mass index.

Additionally, there was little evidence that changes in household purchases of energy and nutrients from HFSS products in Bristol (compared with the counterfactual) varied between specified population subgroups, despite considerable variability by age group, reporting frequency and other differences (OSM T4). However, an increase in sugar from HFSS products purchased in households with a high SES in Bristol compared with the counterfactual was observed (OSM T4). The results also did not indicate changes in mean weekly household OOH purchases of energy, nutrients or packs from HFSS products (OSM T5 and OSM F4).

In sensitivity analyses, including comparison against all three control areas separately (OSM T6, T7 and T8), the increase in mean weekly household purchases of energy from sugary drinks was only evident in comparison to trends in Sheffield. A post hoc analysis excluding sugary drinks from HFSS products showed that without them, the results were comparable to the main findings; the mean change in weekly household energy from HFSS products excluding sugary drinks was 833.5kcal (95% CI −118.2 to 1785.2). Additional adjustment for COVID lockdown periods resulted in minimal changes in the estimates, as were estimates when lockdown periods were excluded from the analysis; changes in mean weekly household purchasing of energy from HFSS products were 875.0 kcal (95% CI −58.7 to 1,808.6) and 936.8 kcal (95% CI −229.9 to 2,103.5), respectively. No evidence of a change in mean weekly household energy was also observed for non-HFSS purchases (187.6 kcal; 95% CI −538.7 to 913.9; data not tabulated).

## Discussion

Our research evaluated the impact of Bristol’s new advertising restriction policy on household purchasing of HFSS products. The study showed no evidence of a change in household purchases of energy from TH or OOH HFSS products in Bristol associated with the policy in the first year following its implementation. There were also no changes in purchasing of total fat, saturated fat, sugar or salt from HFSS products, or evidence of variations in impact across different population subgroups.

### Comparison with other studies

Despite using similar methods, our findings differ from the results on the impact evaluation of the TfL advertising policy in London in 2019, which reported reductions in weekly household purchases of energy (−1,001.0kcal; 95% CI −1546.0 to −456.0), saturated fat (−26.4 g; 95% CI −40.4 to −12.4), fat (−57.9 g; 95% CI −93.7 to −22.1) and sugar (−80.7 g; 95% CI −120.1 to −41.4) from HFSS products.^17^ However, several key differences exist between the TfL policy and Bristol advertisement policy, which could explain their varying impact on purchasing behaviour. For instance, a smaller proportion of advertising sites was affected in Bristol compared with London. The TfL estate consists of 132 060 advertising spaces in trains/stations, over 5000 digital/poster sites at bus shelters and is highly clustered at transport hubs,^25^ whereas in Bristol, the policy only impacted 861 council-owned advertising spaces across 283 bus stops, and digital/poster pavement billboards were not affected due to on-going advertising contracts.^22^ The TfL policy evaluation (which included purchase data from 1970 households during 36-week pre-policy and 44 weeks post-policy) also suggested that when the main food shopper reported using public transport, the reductions in household purchases of HFSS products were larger, which was attributed to a greater probability of advertising exposure. Although data on transport use were not available in our study, travel survey data from both areas for the relevant periods indicated a greater percentage of trips were made using public transport by London residents^26^ compared with Bristol residents.^27^ In addition, the COVID-19 pandemic has resulted in a decrease in bus trip rates, linked to more people working from home.^28^

Collectively, these differences between Bristol and London indicate that the ‘intervention dose’ was much smaller in Bristol compared with London, plausibly contributing to the policy’s lack of impact on HFSS purchasing behaviour. This was further compounded by evidence from another arm of the BEAR study, which analysed the content of bus-stop advertisements in Bristol before and after the policy, and reported that only 11% of bus-stop adverts were not compliant before the policy,^29^ implying that the baseline level of exposure to unhealthy foods/drinks was already fairly low, further minimising the relative change in exposure. This aligns with residents’ perceived advertisement exposure to HFSS products, which was also evaluated in the BEAR study, and for which no change was observed.^30^

Apart from the TfL study, there are no directly comparable studies because previous research has largely evaluated policies targeted at children or more comprehensive national mandatory policies.^10 12 31^ However, in general, the evidence supports the effectiveness of food advertising restriction policies in other settings (broadcast media, digital, transport etc) for reducing purchasing, exposure and/or consumption of unhealthy foods and drinks. An evaluation of Chile’s 2016 national law on food labelling and advertising, which included restrictions on child-directed marketing, showed that household purchases of sugary drinks decreased by 24% compared with the counterfactual.^32^ In addition, evaluations of two policies restricting unhealthy food advertising targeted at children in Quebec^33^ and Singapore^34^ reported a decrease in household fast food expenditures and children’s self-reported consumption of fast food, respectively. Apart from being specifically aimed at children, those policies also covered a much wider range of media than Bristol’s policy.

### Strengths and limitations

This natural experiment evaluation using a CITS analytic method is a robust approach to evaluate the effectiveness of public health interventions and is valuable for informing policy.^35^ We included 17 months prior to the implementation of the policy to capture temporal patterns in consumption more than a year pre-policy. Our study included three different purposively selected control areas, allowing us to incorporate regional and nationwide time-varying confounders into forming the counterfactual, including underlying trends in HFSS purchasing, more unpredictable changes (ie, economic crises/pandemics), as well as seasonal fluctuations. We also adjusted for key confounders, including BMI, SES and seasons and festivals where food/drink purchasing habits often vary. A range of sensitivity analyses was also carried out which supported the robustness of our findings. A further strength is that we analysed OOH purchasing data from a subset of households to account for purchases of food/drinks to be consumed outside of the home (including from shops, restaurants and bars) and which may be more susceptible to impulse purchases after exposure to advertisement. However, these analyses also did not suggest a measurable impact of the policy.

A key limitation is that this is an observational study, so we cannot infer a causal relationship between the advertising restrictions policy and changes, or lack of, in purchasing of HFSS products, or rule out residual confounding. Indeed, residual confounding could be a potential reason why the non-significant effect estimates were positively skewed. Additionally, we did not have information on the travel habits of the household members which could have affected the amount of outdoor advertising exposure within the cities. Another consideration is that the majority of households did not report purchasing every week, with only a third of households reporting over 90% of the study weeks. We assume that irregular reporting was random and non-reporting could partly be due to genuine non-purchasing in certain weeks, that is, due to being on holiday and making larger, more infrequent shops. However, other reasons for missingness and potential bias in the purchase data could be linked to selectively not reporting more unhealthy shopping occasions or food/drink items or not remembering to report smaller shopping occasions.

Nutritional data were not available for the majority of OOH items (84%), so HFSS status had to be based on their market food group that may have introduced non-differential misclassification error. Furthermore, the first 5 months of household purchasing data were collected over two UK COVID-19 lockdown periods, and COVID-19 restrictions have been shown to alter purchasing behaviour, in particular increase total energy,^36^ and could also have reduced exposure to outdoor advertising. However, a sensitivity analysis excluding the first 5 months of purchasing data resulted in similar null findings. It should also be noted that the parallel trends assumption, which is important for CITS inferences, was questionable for certain nutrient and food group analyses. Finally, due to the lower number of households in the subgroup, sensitivity and OOH purchase analyses, it is possible that these were underpowered, so the results from these analyses should be interpreted with caution.

### Policy scope and implications

Bristol’s Advertising and Sponsorship Policy is an important component of Bristol City Council’s long-term ‘One City Plan’, engaging public and private sector organisations to provide a fairer, healthier and safer city, and it aligns with their health in all policies approach.^37^ It was the first city in the UK to implement such a comprehensive outdoor advertising restrictions policy, including widening restrictions beyond HFSS products by including other unhealthy commodities such as gambling. However, the food advertising environment is complex and spans across multiple platforms, including television broadcasting, streaming media and online platforms. Consequently, outdoor advertising only represents a small fraction of total exposure, and to be impactful, unhealthy commodities advertising restriction policies ideally need to target multiple areas within the marketing landscape. The UK’s House of Lords Food, Diet and Obesity Committee report (2024) advises the government to develop a comprehensive new strategy to fix the food system, underpinned by a new legislative framework which includes restricting the advertising of less healthy food across all media.^38^ Therefore, this and similar policies could be seen as part of a wider package of policies and interventions, ideally as primary legislation,^39^ that contribute to a system-wide approach aimed at shifting the population towards healthier dietary habits to reduce NCDs and healthcare costs.

## Conclusion

This natural experiment evaluation found that Bristol’s advertising restrictions policy has not resulted in a measurable impact on household purchasing of unhealthy foods/drinks in the year following the policy. These findings should be interpreted within the scope of the policy, since only a relatively small proportion of outdoor advertising space in Bristol was affected; the reduction in unhealthy food advertising may not have been significant enough on its own to make a measurable impact. As a similar policy in London has been shown to have an effect on curbing purchasing of HFSS products when it impacts on a much larger advertisement environment, this indicates that such advertising restriction policies may need to be extended to cover more outdoor spaces. However, the minimal intervention dose is still unknown and warrants further research.

## Supplementary material

10.1136/bmjph-2025-004137 online supplemental file 1

## Data Availability

Data may be obtained from a third party and are not publicly available.
