# Supplementary material for: Evaluating the changes in household purchases of foods and drinks high in fat, salt and sugar following Bristol’s outdoor advertisement restrictions policy: a quasi-experimental study
Source: BMJ Public Health. 2026 Apr 30;4(2):e004137. doi: 10.1136/bmjph-2025-004137 (PMC13141093; doi:10.1136/bmjph-2025-004137)
Supplement: online supplemental file 1 [file bmjph-4-2-s001.docx]

**Supplementary Material: Evaluating the changes in household purchases of foods and drinks high fat, salt, and sugar (HFSS) following Bristol’s outdoor advertisement restrictions policy: a quasi-experimental study.**

**Authors:**

Buckland G^1^, Sillero-Rejon C^2^, Toumpakari Z^3^, Jago R^1,2^, Yau A^4^, Cummins S^4^, Nobles J^5^, Harding S^2^, Horwood J^2,6^, Nairn A^7^, Hollingworth W^6^, Blake S^8^, de Vocht F^1,2^

**Affiliations:**

^1^Centre for Public Health, Bristol Medical School, University of Bristol, Bristol, UK

^2^NIHR Applied Research Collaboration West (NIHR ARC West), Bristol, UK

^3^Centre for Exercise, Nutrition and Health Sciences, School for Policy Studies, University of Bristol, UK

^4^Population Health Innovation Lab, Department of Public Health, Environments & Society, London School of Hygiene & Tropical Medicine, London, UK

^5^Obesity Institute, School of Health, City Campus, Leeds Beckett University, Leeds, UK

^6^Population Health Sciences, Bristol Medical School, University of Bristol, Bristol, UK

^7^Bristol Hub for Gambling Harms Research, University of Bristol, Bristol, UK

^8^Bristol Medical School, University of Bristol, Bristol, UK

**Supplementary Methods 1(OSM M1):** Methods - Strobe checklist

**Supplementary Methods 2 (OSM M2):** Methods - Kantar’s Worldpanel division Take Home and Out of Home GB household purchasing data collection and analysis and Nutrient Profile Model

**Supplementary Methods 3 (OSM M3):** Methods - Statistical analysis

**Supplementary Table 1 (OSM T1):** Descriptive characteristics of the intervention households (from Bristol) and the control households by area, using Kantar’s Worldpanel division take-home purchasing data.

**Supplementary Table 2 (OSM T2):** Descriptive characteristics of the intervention households (from Bristol) and control households, using Kantar’s Worldpanel division out-of-home purchasing data.

**Supplementary Table 3 (OSM T3):** Unadjusted weekly household mean (SD) energy (kilocalories) purchased from high fat, salt, and sugar (HFSS) products and non-HFSS products pre- and post-intervention in the intervention group and control group**.**

**Supplementary Table 4 (OSM T4):** Stratified analyses for changes in weekly household mean (95% CI) energy and nutrients purchased from high fat, salt, and sugar (HFSS) products, in Bristol (intervention group) compared to the counterfactual, October 2020 to May 2023 (n=1,012), for BMI, number of children in household, age, SES, and reporting frequency.

**Supplementary Table 5 (OSM T5):** Adjusted changes and percentage changes in weekly household mean (95% CI) energy and nutrients purchased from high fat, salt, and sugar (HFSS) products, in Bristol (intervention group) compared to the counterfactual, November 2020 to May 2023 (n = 112) based on Kantar’s Worldpanel division out-of-home purchase data.

**Supplementary Table 6 (OSM T6):** Adjusted changes and percentage changes in weekly household mean (95% CI) energy and nutrients purchased from high fat, salt, and sugar (HFSS) products, in Bristol (intervention group, n=217) compared to South Gloucestershire and Gloucestershire (control, n=280), October 2020 to May 2023, based on Kantar’s Worldpanel division take home purchase data.

**Supplementary Table 7 (OSM T7):** Adjusted changes and percentage changes in weekly household mean (95% CI) energy and nutrients purchased from high fat, salt, and sugar (HFSS) products, in Bristol (intervention group, n=217) compared to Cardiff (control, n=222), October 2020 to May 2023, based on Kantar’s Worldpanel division take home purchase data.

**Supplementary Table 8 (OSM T8):** Adjusted changes and percentage changes in weekly household mean (95% CI) energy and nutrients purchased from high fat, salt, and sugar (HFSS) products, in Bristol (intervention group, n=217) compared to Sheffield (control, n=293), October 2020 to May 2023, based on Kantar’s Worldpanel division take home purchase data.

**Supplementary Figure 1 (OSM F1):** Study flow diagram of households with out-of-home purchasing data

**Supplementary Figure 2 (OSM F2):** Adjusted weekly household mean nutrients (grams) purchased from all HFSS products in Bristol (intervention), Cardiff, Sheffield, Gloucestershire and South Gloucestershire (control), and the counterfactual.

**Supplementary Figure 3 (OSM F3):** Controlled time series graphs for adjusted weekly household mean energy from HFSS food groups purchased in Bristol, control areas and the counterfactual, using take-home data.

**Supplementary Figure 4 (OSM F4):** Controlled time series graphs for adjusted weekly household mean packs of HFSS products purchased in Bristol, control areas and the counterfactual, using out-of-home data.

**Online Supplementary Material: Methods**

**OSM M1: Strobe Checklist**

STROBE Statement—checklist of items that should be included in reports of observational studies

|  | Item No. | Recommendation | Section/Paragraph Number | Relevant text from manuscript |
| --- | --- | --- | --- | --- |
| **Title and abstract** | 1 | (*a*) Indicate the study’s design with a commonly used term in the title or the abstract | Title |  |
|  |  | (*b*) Provide in the abstract an informative and balanced summary of what was done and what was found | Abstract |  |
| Introduction | | | |  |
| Background/rationale | 2 | Explain the scientific background and rationale for the investigation being reported | Introduction/P3-P4 |  |
| Objectives | 3 | State specific objectives, including any prespecified hypotheses | Introduction/P5 |  |
| Methods | | | |  |
| Study design | 4 | Present key elements of study design early in the paper | Methods/Study Design/P5 |  |
| Setting | 5 | Describe the setting, locations, and relevant dates, including periods of recruitment, exposure, follow-up, and data collection | Methods/Participant data/P5 |  |
| Participants | 6 | (*a*) *Cohort study*—Give the eligibility criteria, and the sources and methods of selection of participants. Describe methods of follow-up  *Case-control study*—Give the eligibility criteria, and the sources and methods of case ascertainment and control selection. Give the rationale for the choice of cases and controls  *Cross-sectional study*—Give the eligibility criteria, and the sources and methods of selection of participants | Methods/Participants data/P5 |  |
|  |  | (*b*) *Cohort study*—For matched studies, give matching criteria and number of exposed and unexposed  *Case-control study*—For matched studies, give matching criteria and the number of controls per case |  |  |
| Variables | 7 | Clearly define all outcomes, exposures, predictors, potential confounders, and effect modifiers. Give diagnostic criteria, if applicable | Methods/P5-P6 |  |
| Data sources/ measurement | 8* | For each variable of interest, give sources of data and details of methods of assessment (measurement). Describe comparability of assessment methods if there is more than one group | Methods/P5-P6 |  |
| Bias | 9 | Describe any efforts to address potential sources of bias | Methods/Sensitivity Analyses/P6 |  |
| Study size | 10 | Explain how the study size was arrived at | Methods/P4 |  |

| Quantitative variables | 11 | Explain how quantitative variables were handled in the analyses. If applicable, describe which groupings were chosen and why | Methods/ P5-P6; Supplementary Material/S2 Text/P2-3 |  |
| --- | --- | --- | --- | --- |
| Statistical methods | 12 | (*a*) Describe all statistical methods, including those used to control for confounding | Methods/P5-6 |  |
|  |  | (*b*) Describe any methods used to examine subgroups and interactions | Methods/P6 |  |
|  |  | © Explain how missing data were addressed | Methods/P5-6 |  |
|  |  | (*d*) *Cohort study*—If applicable, explain how loss to follow-up was addressed  *Case-control study*—If applicable, explain how matching of cases and controls was addressed  *Cross-sectional study*—If applicable, describe analytical methods taking account of sampling strategy | Methods/P5-6 |  |
|  |  | © Describe any sensitivity analyses | Methods/P5-6 |  |
| Results | | | | |
| Participants | 13* | (a) Report numbers of individuals at each stage of study—eg numbers potentially eligible, examined for eligibility, confirmed eligible, included in the study, completing follow-up, and analysed | Results/P6/Figure 1 |  |
|  |  | (b) Give reasons for non-participation at each stage | Results/P6 |  |
|  |  | © Consider use of a flow diagram | Fig 1 & Supplementary Figure 1 |  |
| Descriptive data | 14* | (a) Give characteristics of study participants (eg demographic, clinical, social) and information on exposures and potential confounders | Results/P6/Table 1/Supplementary Table 1-3 |  |
|  |  | (b) Indicate number of participants with missing data for each variable of interest | Results/P6/Figure 1 |  |
|  |  | © *Cohort study*—Summarise follow-up time (eg, average and total amount) |  |  |
| Outcome data | 15* | *Cohort study*—Report numbers of outcome events or summary measures over time | Results/P6/Table 2 |  |
|  |  | *Case-control study—*Report numbers in each exposure category, or summary measures of exposure |  |  |
|  |  | *Cross-sectional study—*Report numbers of outcome events or summary measures |  |  |
| Main results | 16 | (*a*) Give unadjusted estimates and, if applicable, confounder-adjusted estimates and their precision (eg, 95% confidence interval). Make clear which confounders were adjusted for and why they were included | Results/P6/ Table 3/ Figure 2&3 |  |
|  |  | (*b*) Report category boundaries when continuous variables were categorized |  |  |
|  |  | © If relevant, consider translating estimates of relative risk into absolute risk for a meaningful time period |  |  |

| Other analyses | 17 | Report other analyses done—eg analyses of subgroups and interactions, and sensitivity analyses | Results/P6/Table 4/Supplementary Tables 4-7/Supplementary Figure 2 |  |
| --- | --- | --- | --- | --- |
| Discussion | | | | |
| Key results | 18 | Summarise key results with reference to study objectives | Discussion/P7-8 |  |
| Limitations | 19 | Discuss limitations of the study, taking into account sources of potential bias or imprecision. Discuss both direction and magnitude of any potential bias | Discussion/P8-9 |  |
| Interpretation | 20 | Give a cautious overall interpretation of results considering objectives, limitations, multiplicity of analyses, results from similar studies, and other relevant evidence | Discussion/P8-9 |  |
| Generalisability | 21 | Discuss the generalisability (external validity) of the study results | Discussion/P10 |  |
| Other information | |  | | |
| Funding | 22 | Give the source of funding and the role of the funders for the present study and, if applicable, for the original study on which the present article is based | Funding Statement P10 |  |

*Give information separately for cases and controls in case-control studies and, if applicable, for exposed and unexposed groups in cohort and cross-sectional studies.

**Note:** An Explanation and Elaboration article discusses each checklist item and gives methodological background and published examples of transparent reporting. The STROBE checklist is best used in conjunction with this article (freely available on the Web sites of PLoS Medicine at http://www.plosmedicine.org/, Annals of Internal Medicine at http://www.annals.org/, and Epidemiology at http://www.epidem.com/). Information on the STROBE Initiative is available at www.strobe-statement.org.

**OMS M2: Kantar’s Worldpanel division Take Home and Out of Home GB Household Purchasing Data and Nutrient Profiling Model**

Kantar Worldpanel maintains a nationally representative live panel of households who provide item-level data on their day-to-day food and beverage purchases. Households within the panel were selected from Bristol (intervention) and three controls areas (Cardiff, Sheffield and South Gloucestershire (SG) and Gloucestershire). The control areas were selected for the following reasons: Cardiff is the nearest city to Bristol, Sheffield is most similar to Bristol in terms of life expectancy indicators in the Public Health Outcomes Framework^1^ and Gloucestershire & SG are two regions within the surrounding area, but with separate governmental local authorities.

Out-of-home (OOH) food and drink purchases were obtained from a subgroup of the households with take-home (TH) purchase data. TH food and drink purchase data is collected by Kantar panel consumers using handheld barcode scanners, while non-barcoded products, such as loose fruits and vegetables, are recorded using bespoke barcodes. OOH consumption was recorded by household individuals who logged meals, snacks and soft drinks bought to be consumed out of home. Each unique food and drink product purchased has a unique purchase id number, which is recorded along with the purchase date, and used to link to nutritional data. Kantar provided information on the total weight and energy, sugar, sodium, saturated fat, protein, and fibre content of each purchase, along with the product’s Nutrient Profiling Model (NPM) score^2^ and HFSS classification (yes/no). When the NPM score and HFSS status of foods/drinks was not provided by Kantar (0.5% of items), these scores and classifications were calculated. For all products, the NPM score calculation was based on the Food Standards Agency methodology ^2^, which is the same method used to determine if the food and non-alcoholic drink advertisements are subject to Bristol advertisement restriction policy. NPM scores are calculated according to product information on energy (kJ), saturated fat (g), sugar (g), sodium (mg), fibre (g), protein (g) and percentage of fruit, vegetables and nuts (%) per 100g of product. Food items that received a score of ≥4 and non-alcoholic drinks that received a score of ≥1 were classified as a HFSS product. Full details of how NPM scores were calculated are outlined in the study protocol (<https://osf.io/7yc9s/>). For OOH data, purchases for individuals within the same household were aggregated together to provide total weekly household out-of-home purchases. The mean weekly household energy (kcal) of nutrients (g) purchased from HFSS products was calculated within weeks with any food of drink purchased.

1. Department of Health and Social Care. Public Health Outcomes Framework (PHOF) . <https://fingertips.phe.org.uk/profile/public-health-outcomes-framework#2> (accessed 21.02.25.

2. Department of Health and Social Care. The nutrient profiling model. London: Department of Health and Social Care. 2011. <https://www.gov.uk/government/publications/the-nutrient-profiling-model> (accessed 01.10.24.

**OSM M3: Statistical Analysis**

A controlled interrupted time series (CITS) design was used to estimate the relative mean change in energy and nutrients from HFSS products in the intervention group (Bristol post-policy period) compared to the counterfactual scenario where the intervention had not occurred (created by extrapolating the pre-intervention trend in the intervention group (74 weeks) combined with the post-intervention changes in the control group (59 weeks)). The CITS model included an interaction term (time x Bristol x intervention) which accounts for the trend in the intervention group (Time × Bristol), the post-intervention period in the intervention group (Intervention × Bristol), the post-intervention trend in the control group (Time × Intervention), and the post-intervention trend in intervention group (Time × Intervention × Bristol).

All CITS models were adjusted for age of main food shopper (continuous), number of adults in household (continuous), number of children in household (continuous), festivals; coded 1 for all weeks including Valentine’s Day, Easter, Halloween, and Christmas and 0 for remaining weeks, season (three separate indicator variables for spring, summer and autumn) coded for each week of the study period as 1 if the week was in the corresponding meteorological season and 0 if not, and socioeconomic position of main food shopper based on National Readership Survey (NRS) occupational social grade (high (A and B), middle (C1 and C2) and low (DE)), BMI of main shopper (<25 kg/m^2^, ≤25 kg/m^2^, missing) and ethnicity of main shopper (white British, white other, non-white, missing).

Cluster-robust standard errors were used to account for clustering of outcomes by household in all models. A gamma distribution was applied to analyse energy and nutrients. The 2-part models were used to estimate the mean weekly household energy and nutrients purchased from HFSS products and used pairwise comparisons to test the difference in marginal means in the intervention group compared to the counterfactual in the post-intervention period. The percentage change in average marginal effects compared to the counterfactual was estimated using linear comparisons of parameters. These percentage changes (reductions or increases) are in the context of any secular changes in HFSS purchases in both the intervention and control areas. OOH food and drink items without nutritional information were analysed as change in packs of HFSS products.

The CITS analyses were repeated including each the control areas separately. Stratified analyses were used to assess if any potential changes varied by BMI category of main shopping (overweight/obese vs not overweight/obese), presence of children in the household (yes vs no), socioeconomic status (low, medium and high) and age of main food shoppers (<50 years vs ≥50 years).

**Online Supplementary Material: Tables**

**OSM T1:** Descriptive characteristics of the intervention households (from Bristol) and the control households by area, using Kantar’s Worldpanel division take-home purchasing data.

**OSM T2:** Descriptive characteristics of the intervention households (from Bristol) and control households, using Kantar’s Worldpanel division out-of-home purchasing data.

**OSM T3.** Unadjusted weekly household mean (SD) energy (kilocalories) purchased from high fat, salt, and sugar (HFSS) products and non-HFSS products pre- and post-intervention in the intervention group and control group, using take-home Kantar’s Worldpanel division take home purchase data**.**

**OSM T4.** Stratified analyses for changes in weekly household mean (95% CI) energy and nutrients purchased from high fat, salt, and sugar (HFSS) products, in Bristol (intervention group) compared to the counterfactual, October 2020 to May 2023 (n=1,012), for BMI, number of children in household, age, SES, and reporting frequency.

Abbreviations: HFSS - High fat, sugar and salt products. Weekly household mean purchases were estimated from a controlled interrupted time series 2-part model: part 1 (logit) and part 2 (generalised linear model), with gamma distribution for energy and nutrients and negative binomial distribution for packs. Models were adjusted for festivals, season, number of adults in household, number of children in household, age, ethnicity, BMI of main food shopper and socioeconomic position of main food shopper. Cluster-robust standard errors were used. Household-week observations where households did not report any food and drink purchases that week were dropped. Data period = October 2020 to May 2023. *BMI relates to main household shopper and is missing for N=185.

**OSM T5.** Adjusted changes and percentage changes in weekly household mean (95% CI) energy and nutrients purchased from high fat, salt, and sugar (HFSS) products, in Bristol (intervention group) compared to the counterfactual, November 2020 to May 2023 (n = 112) based on Kantar’s Worldpanel division out-of-home purchase data.

**OSM T6.** Adjusted changes and percentage changes in weekly household mean (95% CI) energy and nutrients purchased from high fat, salt, and sugar (HFSS) products, in Bristol (intervention group, n=217) compared to South Gloucestershire and Gloucestershire (control, n=280), October 2020 to May 2023, based on Kantar’s Worldpanel division take home purchase data.

**OSM T7:** Adjusted changes and percentage changes in weekly household mean (95% CI) energy and nutrients purchased from high fat, salt, and sugar (HFSS) products, in Bristol (intervention group, n=217) compared to Cardiff (control, n=222), October 2020 to May 2023, based on Kantar’s Worldpanel division take home purchase data.

**OSM T8:** Adjusted changes and percentage changes in weekly household mean (95% CI) energy and nutrients purchased from high fat, salt, and sugar (HFSS) products, in Bristol (intervention group, n=217) compared to Sheffield (control, n=293), October 2020 to May 2023, based on Kantar’s Worldpanel division take home purchase data.

**Online Supplementary Material: Figures**

**OSM F1:** Study flow diagram detailing eligibility and inclusion of households and household weekly out-of-home purchasing data from Kantar’s Worldpanel division.

**
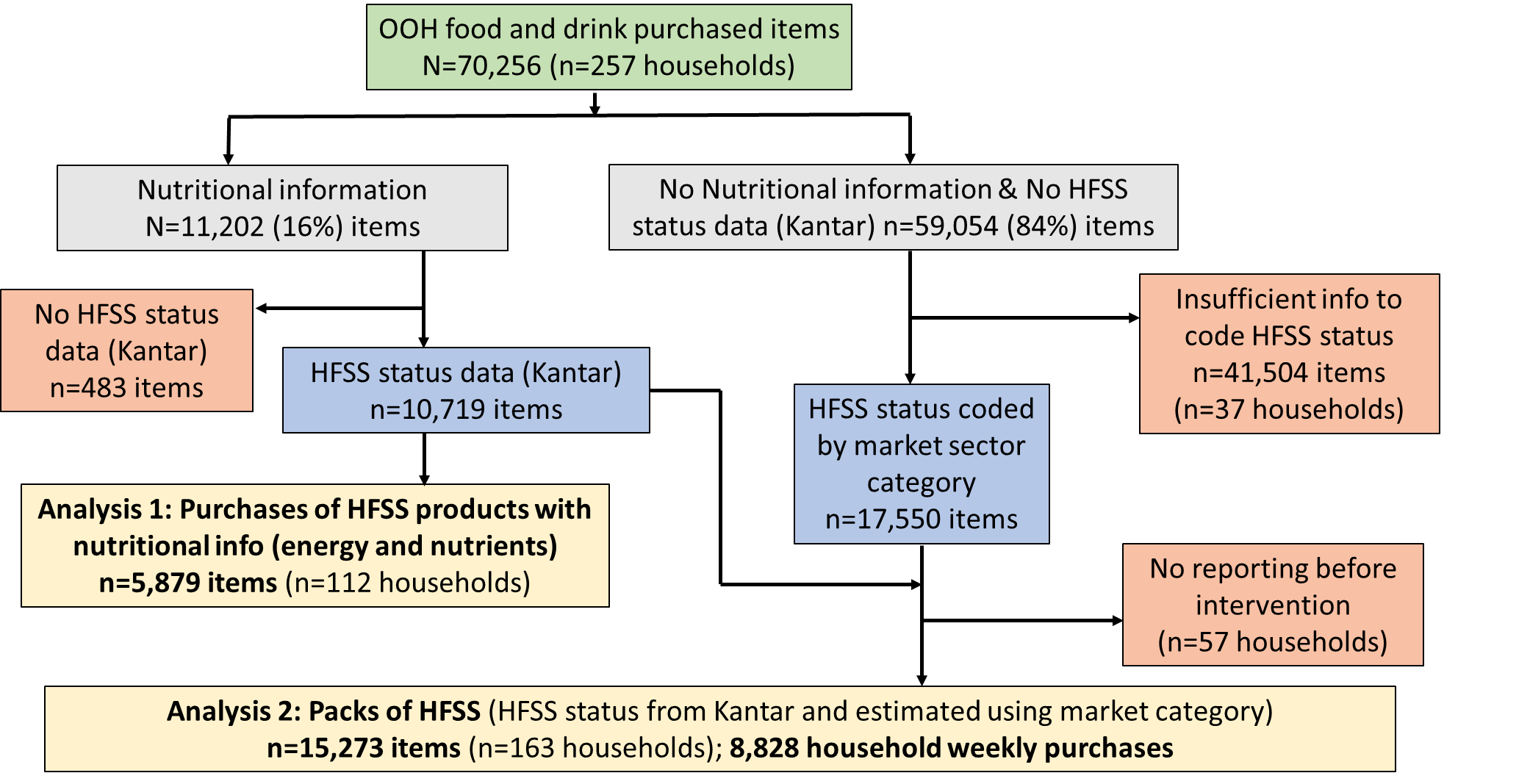
**

**OSM F2:** Adjusted weekly household mean nutrients (grams) purchased from all HFSS products in Bristol (intervention), Cardiff, Sheffield, Gloucestershire and South Gloucestershire (control), and the counterfactual. Vertical line = date of intervention implementation. Gray shaded area = period up until end of last main Covid lockdown staged exit (end of March 2021). Blue x and red + indicate data points for Bristol and control areas, respectively. Data period = October 2020 to May 2023. Spikes represent festival weeks included in the adjusted models. Numerical values linked to this graph are detailed in Table 3 in main paper.

**
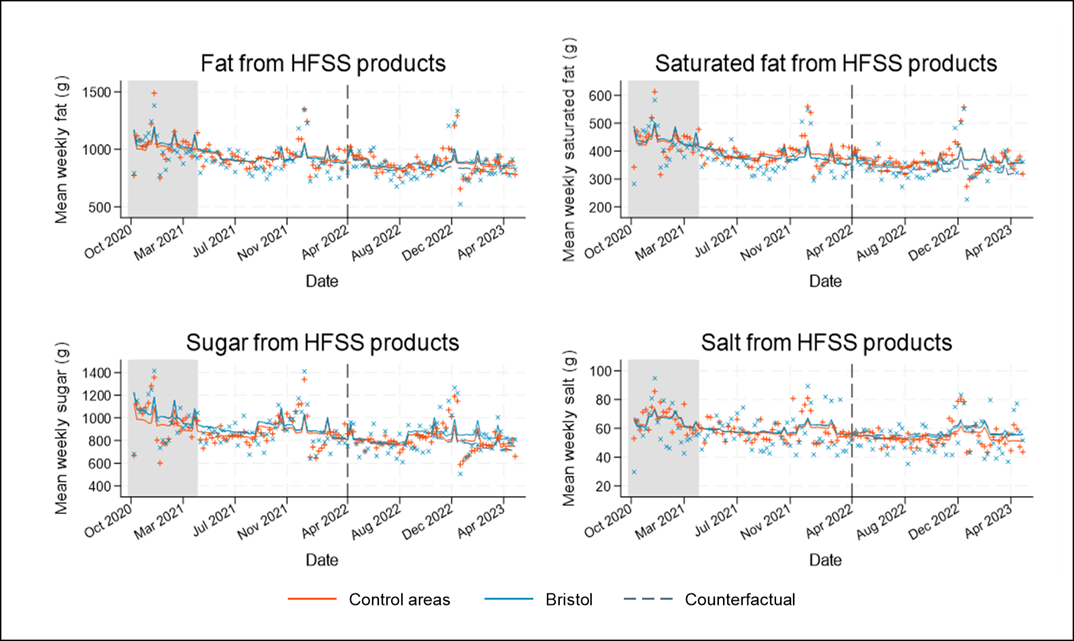
**

**OSM F3:** Adjusted weekly household mean energy purchased (kcal) from HFSS product food groups in Bristol (intervention), controls areas (Cardiff, Sheffield, Gloucestershire and South Gloucestershire) and the counterfactual, based on Kantar’s Worldpanel division take home purchase data.

Vertical line = date of intervention implementation. Gray shaded area = period up until end of last Covid lockdown staged exit (end of March 2021). Blue x and red + indicate data points for Bristol and control areas. The counterfactual was estimated by extrapolating the preintervention trend in Bristol and incorporating the post-intervention changes in the control areas. Models were adjusted for festivals, season, number of adults in household, number of children in household, age, socioeconomic position and BMI category of main food shopper and ethnicity. Household-week observations where households did not report any food and drink purchases that week were dropped. Data period = October 2020 to May 2023. Spikes represent festival weeks included in the models.

**OSM F4:** Adjusted weekly household mean packs (kcal) of HFSS products purchased in Bristol (intervention) households, control households (Cardiff, Sheffield, Gloucestershire and South Gloucestershire) and the counterfactual, based on Kantar’s Worldpanel division out-of-home purchase data.

Abbreviations: OOH - Out-of-home
